# Beyond Dairy: Consumer Perceptions and Beliefs About Dairy Alternatives—Insights from a Segmentation Study

**DOI:** 10.3390/foods15010077

**Published:** 2025-12-26

**Authors:** Sylwia Żakowska-Biemans

**Affiliations:** Department of Food Market and Consumer Research, Institute of Human Nutrition Sciences, Warsaw University of Life Sciences, Nowoursynowska 159c, 02-776 Warsaw, Poland; sylwia_zakowska_biemans@sggw.edu.pl

**Keywords:** plant-based dairy alternatives (PBDAs), consumer segmentation, pro-dairy justifications, avoidance/dissociation, familiarity/awareness, consumer beliefs, Poland

## Abstract

Increasing consumption of plant-based alternatives is promoted to reduce the environmental impact of food systems, yet adoption remains limited. The aim of this study was to identify distinct consumer segments and examine differences in their perceptions, consumption habits, and trial intentions concerning plant-based dairy alternatives (PBDAs). Conceptually, it advances PBDAs segmentation by jointly incorporating pro-dairy justifications, avoidance of animal-origin considerations, and self-reported PBDAs familiarity, capturing psychological defence mechanisms alongside knowledge-related influences on adoption. Data were collected in a nationwide cross-sectional CAWI survey of 1220 Polish adults responsible for household food purchasing, stratified and quota-matched by gender, age, region, and settlement size. Factor analysis of the segmenting variables was conducted using principal component analysis with varimax rotation, followed by two-step cluster analysis. Alternative cluster solutions were compared using the Bayesian Information Criterion based on the log-likelihood (BIC-LL). The selected five-cluster solution showed acceptable to good clustering quality, as indicated by silhouette-based measures of cohesion and separation. Given the cross-sectional CAWI design and reliance on self-reported measures, the findings do not allow causal inference and should be interpreted as context-specific to the Polish, dairy-centric food culture. Cluster analysis identified five segments that differed in PBDA-related beliefs, product image evaluations, consumption patterns, and trial intentions. PBDA-oriented segments, comprising a dairy-critical segment and a dual-consumption segment, exhibited higher perceived familiarity and stronger ethical and environmental concerns and showed greater PBDA use and willingness to try new products. The dual-consumption segment reported the highest use and trial readiness. In contrast, resistant segments showed stronger dairy attachment, lower perceived familiarity, and more sceptical evaluations of PBDAs’ healthfulness, naturalness, and sensory appeal, and rarely consumed plant-based alternatives. The findings highlight substantial heterogeneity in how Polish dairy consumers perceive PBDAs, emphasising the importance of segment-specific approaches for communication and product development. Tailored strategies can help address the diverse motivations and barriers of consumers, supporting a dietary shift toward more plant-based options.

## 1. Introduction

Plant-based dairy alternatives (PBDAs) demonstrate substantial market growth worldwide, driven by a complex interplay between environmental, health, and socio-economic factors that jointly reshape consumer preferences and purchasing behaviours [1,2,3,4,5]. The growing evidence of dairy farming’s environmental impact, combined with increasing climate awareness, has positioned plant-based alternatives as more sustainable options across multiple environmental dimensions [6,7]. Increased consumption of dairy product alternatives is perceived as contributing to the transformation of agri-food systems in accordance with sustainability principles, but the success of this process largely depends on a multidimensional understanding of consumer decision-making mechanisms underlying behaviours related to dairy alternatives adoption [8]. Consumer adoption remains a complex process shaped by multiple interconnected factors, including personal motives, perceptions, and beliefs. Health considerations serve as primary drivers for initial product trial and continued consumption [3,9,10]. These include perceived digestibility benefits, accommodation of dietary restrictions such as lactose intolerance, and broader concerns related to cholesterol and saturated fat content.

Environmental concern is identified as one of the most important motives for choosing alternatives to milk and dairy products [1,11]. However, these issues should be considered in the broader context of impact on the planet and climate, considering raw material and local context [12]. The environmental footprint associated with sourcing raw materials for dairy alternatives varies significantly and, under high demand, may intensify resource use, resulting in negative externalities [13]. Beyond environmental consciousness, animal welfare values also function as significant motivators, particularly among younger consumers who demonstrate heightened sensitivity to sustainability issues [14]. Adolescents and young adults exhibit a particular responsiveness to these ethical drivers, often perceiving PBDAs consumption as aligning with their environmental values and personal identity formation [10].

Plant-based dairy alternatives constitute a heterogeneous product category developed to replace conventional dairy across various consumption contexts, including beverages (e.g., soy, oat, and almond-based drinks), fermented products (yoghurt, kefir alternatives), and cheese substitutes [15,,16,17,18]. The diversity of raw materials and processing methods results in significant variability in nutritional profiles, sensory attributes, and consumer acceptance [19,20,21]. Importantly, compositional evidence reveals substantial variability in protein quality and micronutrient profiles across different plant-based dairy alternative types and markets, with nutritional characteristics often diverging from those of dairy and largely influenced by fortification practices [15,22,23,24,25]. While soy-based alternatives may approach dairy in protein quality, many other plant-based products show lower protein content and rely on fortification strategies to supply key micronutrients such as calcium, vitamin D, vitamin B12, and iodine [16,26].

Plant-based milks and other dairy alternatives are increasingly used in cooking and baking [27]. Products like soy milk and almond milk are valued for their versatility and ability to mimic the functional properties of dairy milk in recipes [28,29,30].

Price sensitivity and retail availability also shape purchasing decisions, particularly in regions where product variety remains limited. Additionally, use-case considerations play a crucial role: consumers assess PBDAs’ performance in specific applications such as coffee, cereal, cooking, and baking, and these practical experiences often determine whether products become part of regular purchasing routines or are only bought occasionally [31]. Sensory attributes, including taste, texture, and aroma, play a crucial role in determining consumer acceptance of dairy alternatives. Achieving the desired sensory qualities in plant-based products remains a significant challenge, mainly due to the inherent differences between plant-based ingredients and their animal-based counterparts. [32,33]. Consumers often find plant-based milks to have off-flavours or less desirable textures compared to dairy milk [34]. Plant-based cheese alternatives are often criticised for sensory shortcomings, particularly in flavour and texture [35]. Beyond sensory shortcomings, significant concerns have been raised regarding the nutritional value of plant-based dairy alternatives. These concerns center on lower protein quality and content, reduced levels of essential micronutrients such as calcium, vitamin B12, and iodine, and the variable effectiveness of fortification strategies, which together can impact the overall nutritional adequacy when compared to traditional dairy products [15,36].

In Poland, milk consumption remains relatively stable, although its structure is changing. Polish food culture strongly favours dairy, with milk consumption embedded in family and social practices [37,38]. Older adults and children consume milk more frequently, whereas younger generations, especially those residing in large cities, are increasingly opting for plant-based beverages as a substitute for milk [39,40]. Curiosity, perceived health benefits, and social influence are key motives for trying plant-based dairy alternatives, while strong attachment to traditional dairy and daily habits remain significant barriers to substitution [41]. Sensory concerns—particularly regarding taste and texture—are frequently reported, with some variants considered less palatable than their dairy counterparts [40,42]. Moreover, consumers express uncertainty about the nutritional value, degree of processing, and “naturalness” of plant-based alternatives, which may undermine their perceived health and sustainability benefits [41].

Understanding the psychological diversity underlying consumer attitudes toward dairy and its plant-based alternatives is increasingly critical for food industry stakeholders, policymakers, and researchers. Dietary choices are not merely behavioural; they are shaped by a complex interplay of cognitive rationalisations, emotional attachments, and ethical values. As the market for plant-based alternatives expands, capturing this multidimensional aspect through segmentation studies is crucial for effective product development, marketing, and public health interventions [18]. Previous research on plant-based dairy alternatives has largely focused on socio-demographic predictors, general attitudes toward sustainability or health, and product-specific evaluations. While informative, such approaches often overlook the psychological mechanisms that sustain attachment to dairy despite increasing awareness of environmental and ethical concerns. To address this gap, the present study concentrates on three theoretically grounded dimensions: pro-dairy justifications, avoidance of animal-origin considerations, and self-reported familiarity with plant-based dairy alternatives. Together, these constructs capture cognitive rationalisations, defence strategies, and perceived awareness that operate upstream of product evaluation and purchasing behaviour, and are therefore particularly relevant for understanding uneven PBDAs adoption. Addressing this gap is particularly pertinent in dairy-centric food cultures such as Poland, where dairy consumption is deeply embedded in everyday practices and cultural norms, while the market for plant-based dairy alternatives remains comparatively underdeveloped. This context provides an advantageous setting for examining how psychological defence mechanisms and perceived familiarity collectively influence openness to, or resistance against, plant-based dairy alternatives, beyond what can be deduced from attitudinal or demographic segmentation alone.

Therefore, the primary objective of this study was to enhance understanding of the factors that influence consumers’ adoption of plant-based dairy alternatives. The specific aims were to (1) identify distinct consumer segments based on their pro-dairy justifications, avoidance of the animal origin of dairy, and familiarity with plant-based dairy alternatives; and (2) characterise these segments in terms of their perceptions and beliefs of plant-based dairy alternatives, their consumption of dairy and its plant-based counterparts, the conditions under which they would be willing to try plant-based dairy alternatives, and their socio-demographic characteristics.

## 2. Materials and Methods

### 2.1. Participants

This study was conducted on a nationwide sample (*n* = 1220) using selection criteria matched to the population distributions in Poland for gender, age, place of residence, and region, employing stratified random sampling. Eligibility was restricted to individuals aged 18 years and above who were responsible or co-responsible for decision-making and food purchases within their households. Data were collected using the Computer-Assisted Web Interview (CAWI) technique. The process was administered by a professional social research agency operating in compliance with the standards of the European Society for Opinion and Marketing Research (ESOMAR) and holding the Polish Interviewer Quality Control Program (PKJPA) certificate for online research. All procedures complied with the General Data Protection Regulation (GDPR; Regulation (EU) 2016/679 of the European Parliament and of the Council of 27 April 2016). The study adhered to the principles of the Declaration of Helsinki. After screening for eligibility, respondents were informed beforehand about the study aims, procedures, and their right to withdraw at any point. They could either exit without completing the questionnaire or proceed to the next page, provide informed consent, and continue to the main questionnaire. All data collected was anonymised and processed in a non-identifiable format.

### 2.2. Measures

#### 2.2.1. Segmentation Basis

To capture the psychological underpinnings of consumer attitudes toward dairy products and PBDAs, consumer segmentation was based on three theoretically grounded constructs: pro-dairy justifications, dissociation/avoidance of the animal origin of dairy, and familiarity with plant-based alternatives. This multidimensional approach recognises that choices related to dairy products are shaped not only by preferences and habits, but also by moral reasoning, individual knowledge, and psychological defence mechanisms [4,43]. The first construct, pro-dairy justifications, reflects cognitive and emotional rationalisations that support continued dairy consumption. It was measured using five items adapted from the Meat Justification Scale [17] and rephrased for the dairy context. Respondents rated their agreement with the following statements: (1) “We need milk and dairy products for healthy nutrition,” (2) “Dairy products are too tasty to worry about what critics say,” (3) “I enjoy eating/drinking dairy so much that I could never give it up,” (4) “Animals don’t really suffer when they are used for milk production,” and (5) “Milk and dairy products are produced in a way that minimises animal pain and discomfort.” Responses were recorded on a 7-point Likert scale (1 = strongly disagree, 7 = strongly agree). These items were loaded onto a single factor labelled pro-dairy justifications, which showed good internal consistency (Cronbach’s α = 0.82).

The second construct, dissociation and avoidance, assessed psychological distancing from the animal origin of dairy. It included four items: (1) “When I look at dairy products, I try not to associate them with animals,” (2) “I don’t like to think about where the dairy products I consume come from,” (3) “I try not to think about what happens in the dairy industry,” and (4) “I would have a problem visiting a dairy farm.” These items formed a single factor labelled dissociation/avoidance, with acceptable internal consistency (Cronbach’s α = 0.61).

The third construct, familiarity with plant-based alternatives, captured respondents’ self-reported perceived familiarity/awareness of plant-based alternatives and the rationale for their development. It was assessed through three items: (1) “I know the advantages and disadvantages of plant-based alternatives to animal products,” (2) “I know what plant-based alternatives to animal products mean,” and (3) “I know why the production of plant-based alternatives to animal products should be developed.” These items, also rated on the 7-point Likert scale, were loaded onto one factor labelled plant-based familiarity, with satisfactory reliability (Cronbach’s α = 0.75). These three constructs were selected as the basis for segmentation because they capture complementary psychological processes underlying dietary continuity and change. Pro-dairy justifications reflect mechanisms that legitimise continued dairy consumption, while avoidance and dissociation capture strategies aiming to manage moral or emotional discomfort related to animal-derived foods. Familiarity with PBDAs represents a perceived awareness that conditions openness to experimentation and adoption. Unlike socio-demographic or purely attitudinal variables, these dimensions enable the examination of resistance and openness to plant-based alternatives simultaneously within a single segmentation framework.

#### 2.2.2. Profiling Factors

To describe and compare the resulting segments, four sets of profiling variables were used. The first set captured innovation-adoption characteristics of plant-based dairy alternatives and was adapted from [31]. Perceived compatibility was measured with three items stating that plant-based dairy alternatives are attractive in daily life, fit well into respondents’ usual eating habits, and are generally unproblematic to consume. Perceived relative advantage was assessed with three statements that these products are environmentally friendly, help to reduce food insecurity, and contribute to mitigating climate change. Together, these six items showed good internal consistency (Cronbach’s α = 0.90). Trialability was measured using three items that expressed a desire to try plant-based dairy alternatives before purchase, to receive free samples, and to obtain information or guidance on how best to prepare and consume them (Cronbach’s α = 0.87).

The third set of profiling variables reflected the perceived product image of plant-based dairy alternatives, assessed with ten 7-point semantic differential items (unhealthy–healthy, unfavourable–favourable for the environment, traditional–modern, low–high nutritional value, low-processed–highly processed, non-innovative–innovative, unnatural–natural, unpalatable–tasty, hard to access–easy to access, cheap–expensive). After recoding where necessary, higher scores indicated a more positive, modern image of plant-based dairy alternatives. Internal consistency for this scale was modest but acceptable for descriptive profiling purposes (Cronbach’s α = 0.61).

Finally, the segments were also profiled based on declared consumption of dairy and plant-based dairy alternatives. Respondents reported their usual frequency of intake of cow’s milk, fermented dairy drinks (yoghurt, kefir), curd/cottage cheeses and desserts, as well as plant-based milk, yoghurt/kefir, and cheese alternatives, using a six-point frequency scale ranging from “never” to “several times a day”.

### 2.3. Data Analysis

Data were analysed using descriptive statistics, parametric (ANOVA) and non-parametric tests (chi-square), as well as multivariate analysis (factor analysis) and two-step cluster analysis. In the first stage, factor analysis was conducted on the segmenting variables using the principal component analysis (PCA) method with varimax rotation. Subsequently, the two-step clustering method was applied to identify consumer segments. Cluster solutions were compared using the Bayesian Information Criterion based on the log-likelihood (BIC-LL). The BIC-LL reached its lowest value for the five-cluster solution; therefore, it was adopted as the final one. Cluster quality was additionally assessed using the silhouette measure, which indicated acceptable to good internal cohesion and separation of the five-cluster solution. After the clusters were defined, the next step was to profile these segments. This analysis was conducted using chi-square cross-tabulation and ANOVA, followed by post hoc Scheffé tests to compare the mean scores. The collected data were analysed using the IBM SPSS Statistics v. 29 statistical software package, Armonk, NY, USA: IBM Corp.

## 3. Results

### 3.1. Factor Analysis Results

An exploratory factor analysis was conducted on 12 items that covered pro-dairy justification, avoidance, dissociation, and familiarity. The Kaiser–Meyer–Olkin (KMO) test yielded a value of 0.783, indicating good sampling adequacy for factor analysis. Bartlett’s test of sphericity was significant (χ^2^ = 3984.97, df = 66, *p* < 0.001), confirming that the correlation matrix was factorable and appropriate for factor extraction. Principal component analysis with Varimax rotation identified three factors, each with an eigenvalue greater than 1.0, accounting for 58.65% of the total variance (Table 1).

The three-factor solution demonstrated satisfactory interpretability, in accordance with Kaiser’s criterion for eigenvalue retention. Factor 1: Dairy Attachment and Necessity (accounting for 25.46% of the variance) captured strong positive attitudes toward traditional dairy consumption, encompassing beliefs about its nutritional value, taste preferences, and justifications related to animal welfare. This dimension reflects both emotional attachment and the perceived indispensability of dairy products. Factor 2: Plant-Based Knowledge and Familiarity (17.19% of the variance) represented consumers’ self-reported knowledge and awareness of plant-based alternatives. This factor indicates cognitive understanding of dairy substitutes and their broader implications. Factor 3: Avoidance and Dissociation (16.01% of the variance) reflected psychological distancing from the realities of dairy production. This dimension encompasses cognitive avoidance and emotional dissociation strategies aimed at minimising discomfort associated with animal-derived food sources.

### 3.2. Sample and Clusters Profile

Table 2 presents the demographic profile of the overall sample and the distribution of respondents across the five identified clusters. The gender split was nearly equal, with 50.6% identifying as women and 49.4% as men. Participants represented all age groups between 18 and 70 years: 9.5% were aged 18–24, 17.8% were 25–34, 23.5% were 35–44, 20.5% were 45–54, 17.3% were 55–64, and 11.5% were 65–70 years old.

In terms of education, 7.4% of respondents had completed primary education, 26.3% held vocational (basic) qualifications, 37.9% had secondary education, and 28.4% possessed higher education (Bachelor’s, Engineer, or Master’s degree). Geographical distribution varied: 40.0% lived in rural areas, 13.1% in towns with up to 20,000 residents, 10.7% in towns of 20,000–50,000, 16.7% in cities of 50,000–200,000, and 19.5% in cities exceeding 200,000 residents; 13.1% chose not to disclose their place of residence. Regarding self-assessed economic status, 59.4% described their income as sufficient for daily needs but requiring savings for larger expenses, 13.3% reported modest living conditions requiring economizing, and 4.0% indicated serious financial hardship. In contrast, 18.6% reported living comfortably without major savings needs, and 1.4% rated their standard of living as very good with access to some luxuries (3.3% expressed no opinion).

Cluster 4 constituted the largest segment of the sample at 38.5%, followed by Cluster 2 with 18.5% and Cluster 1 with 17.4%. Cluster 5 represented 14.2% of respondents, while Cluster 3 was the smallest segment at 11.4%. The chi-square test showed that cluster membership was significantly associated with gender, age, and educational level, but not with settlement size (Pearson’s χ^2^(16) = 18.37, *p* = 0.303).

Gender distribution differed markedly between clusters (Pearson’s χ^2^(4) = 33.47, *p* < 0.001). Cluster 3 was predominantly female (65.7%), whereas Cluster 5 was predominantly male (63.0%). Cluster 2 also had a higher proportion of men (57.1%), while Clusters 1 and 4 exhibited near parity, with a slight predominance of women (53.6% and 53.5%, respectively).

Age composition also varied significantly across clusters (Pearson’s χ^2^(20) = 120.65, *p* < 0.001), indicating distinct demographic profiles. Cluster 2 was the oldest segment, with relatively high proportions of respondents aged 45–54, 55–64, and 65–70 years, and comparatively few in the youngest categories (18–24 and 25–34 years). Cluster 5 likewise skewed towards older respondents, with large shares in the 45–54 and 55–64 age groups and very small representation of the youngest adults. By contrast, Clusters 1, 3, and 4 were relatively younger or middle-aged, with higher proportions in the 25–34 and 35–44 categories and lower shares of respondents aged 65–70 years.

Educational attainment differed significantly between clusters (Pearson’s χ^2^(12) = 38.33, *p* < 0.001). Cluster 3 was the most highly educated segment, with the highest proportion reporting tertiary education and the lowest share with vocational education. Cluster 5 showed a more vocational profile, combining the highest proportion with vocational education and a relatively high share with only primary education, alongside a lower proportion with secondary education. Cluster 2 was characterised by a relatively high prevalence of secondary education, whereas Cluster 4 combined comparatively high vocational participation with lower tertiary attainment.

Perceived household financial situation differed significantly across clusters (Pearson’s χ^2^(20) = 36.33, *p* = 0.014). In all clusters, the most common response was that income covers basic needs but requires saving for larger purchases, although the proportion of respondents endorsing this category ranged from just over half in Cluster 1 to about two-thirds in Cluster 5. Cluster 1 showed a polarised profile, with many reporting severe financial difficulties and a high need to economise, but also a sizeable group living comfortably without issues saving. Cluster 2 was the most financially advantaged segment, with the lowest share reporting extreme hardship and the highest proportion indicating they can afford larger expenses without special savings, as well as one of the highest shares reporting a very good material situation. Cluster 3 also displayed relatively positive self-assessments, with the lowest proportion needing to cut back significantly and a comparatively high share reporting ease with larger expenditures, although it also had the highest proportion of respondents expressing uncertainty about their financial situation. Cluster 4 seemed more economically constrained, with the highest proportion experiencing severe difficulty, a relatively high share needing to economise, and the lowest proportion able to afford larger expenditures without saving. Cluster 5 was characterised by a mainly “middle” profile, with the greatest concentration in the “basic needs covered but saving required for larger purchases” category, low levels of extreme hardship, and no respondents reporting a very affluent situation.

### 3.3. Cluster Profiles Based on Segmentation Variables

Table 3 presents mean scores for the total sample and for each of the five identified clusters on the items used as segmenting variables, capturing pro-dairy justifications, familiarity with plant-based alternatives, and dissociation/avoidance.

Cluster 1 demonstrated a strong commitment to dairy consumption, with high agreement that milk is essential for a healthy diet and that dairy products are challenging to give up. Although they generally scored slightly lower than Cluster 2, they were significantly higher than the more moderate Clusters 3 and 4. What distinguishes this group is the combination of high attachment with marked cognitive and emotional defensiveness. They reported the highest levels of avoiding thoughts about the dairy industry, avoiding thinking about the origin of dairy products, not wanting to associate dairy products with animals, and having difficulty visiting a dairy farm; on these items, their means were significantly higher than in Clusters 2–5. They also tended to believe that milk production prevents animal pain more than Clusters 3 and 4. In contrast to their defensive stance, they rated their familiarity with PBDAs as high: across the three items, their scores were comparable to Cluster 3 and often higher than Cluster 2 (especially for the rationale to develop PBDAs), and significantly higher than Clusters 4 and 5. Overall, this segment appears to consist of relatively well-informed but psychologically defensive dairy enthusiasts. As a result, a name was proposed for this segment—“Defensive but informed dairy enthusiasts”.

Cluster 2 showed the strongest and most consistent attachment to dairy. They scored highest on statements such as “I could never give up dairy,” “We need milk for a healthy diet,” and “Dairy is too tasty to care about critics.” They also strongly minimised animal suffering, with mean scores significantly above the more critical Cluster 3 and the more moderate Cluster 4. Levels of cognitive dissonance were the lowest in this group—they rarely avoided thinking about the dairy industry, had little difficulty associating products with animals, and were most comfortable visiting a dairy farm. While their PBDAs familiarity was relatively high, they scored significantly lower than Cluster 3 on the item measuring motivation to support plant-based alternatives. Based on these characteristics, this segment was labelled “Unquestioning Dairy Traditionalists”.

Cluster 3 was the most sceptical of dairy and the most supportive of PBDAs. They reported the lowest scores across all items related to dairy attachment and perceived necessity—significantly less likely than any other cluster to agree that dairy is essential for a healthy diet, difficult to give up, or too tasty to question. They also strongly rejected justifications related to animal welfare, scoring the lowest on both items concerning animal pain and suffering. Unlike Cluster 1, they did not cope with these concerns through avoidance; they showed the least tendency to dissociate dairy products from animals or to avoid thinking about the dairy industry, with scores significantly below those of Cluster 1 and often lower than those of Cluster 4 and 5. Their reported familiarity with PBDAs was high, including strong agreement with the rationale for developing PBDAs. This cluster comprises a small yet highly informed group that criticises traditional dairy and supports dietary change. Based on these traits, this segment was named “PB-Oriented Dairy Critics”.

Cluster 4 demonstrated moderate positions across all key dimensions. Their endorsement of dairy attachment and necessity statements was significantly lower than in Clusters 1 and 2, but higher than in Cluster 3. Responses to animal welfare items reflected ambivalence, with scores falling between the more accepting Clusters 1, 2, and 5, and the critical Cluster 3. Levels of cognitive dissonance were also intermediate—higher than in Cluster 2, but lower than in Cluster 1. Familiarity with PBDAs was moderate, significantly lower than Clusters 1–3, but still consistently higher than Cluster 5. Overall, this segment represents a pragmatic consumer group with generally favourable views of dairy and cautious openness to plant-based alternatives. Based on these characteristics, this segment was labelled “Moderate Dairy Consumers”.

Cluster 5 shared a generally positive orientation toward dairy, similar to Clusters 1 and 2. They strongly agreed that milk is essential for a healthy diet and that dairy products are too enjoyable to be influenced by criticism, with mean scores significantly higher than those of the more sceptical Clusters 3 and 4, and comparable to those of Clusters 1 and 2. Their responses on cognitive dissonance items, such as avoiding thoughts about the dairy industry, dissociating products from animals, and discomfort with farm visits, were intermediate: significantly higher than the relaxed Cluster 2 but lower than the strongly defensive Cluster 1. On animal welfare items, they leaned toward a pro-dairy stance, scoring above Cluster 3 (and often Cluster 4), though somewhat below Cluster 2. What clearly distinguishes this group is their very low familiarity with PBDAs; across all three familiarity-related items, they scored significantly lower than all other clusters. This suggests a segment with strong attachment to dairy, coupled with limited awareness or understanding of plant-based alternatives. Based on these characteristics, this segment was labelled “Dairy-Oriented PB-Uninformed”.

### 3.4. Beliefs About Plant-Based Dairy Alternatives

To further characterise the identified segments, general beliefs about PBDAs were examined. Respondents evaluated PBDAs based on their everyday appeal, how well they align with typical eating habits, perceived ease of consumption, and their beliefs about their environmental advantages, such as reducing food insecurity and combatting climate change (Table 4).

The five identified consumer segments ranged from PB-Oriented Dairy Critics and Defensive but Informed Dairy Enthusiasts, who expressed the most favourable attitudes towards PBDAs, through Moderate Dairy Consumers occupying a moderate position, to the more sceptical Unquestioning Dairy Traditionalists and Dairy-Oriented PB-Uninformed Consumers, who showed the least acceptance and awareness of PBDAs.

Cluster 1 (Defensive but Informed Dairy Enthusiasts), despite a strong attachment to traditional dairy, holds a clearly positive view of PBDAs. They rated PBDAs as highly attractive and well-integrated into daily routines, with mean scores significantly higher (*p* < 0.05) than those of Clusters 2, 4, and 5, and statistically comparable to Cluster 3. They also perceived PBDAs consumption as unproblematic and expressed strong agreement with sustainability-related benefits, including environmental friendliness, contributions to food security and climate change mitigation —again with scores significantly higher than Clusters 2 and 5, and more favourable than Cluster 4.

Cluster 2 (Unquestioning Dairy Traditionalists) consistently show scepticism towards PBDAs. They rated PBDAs as the least appealing and least compatible with their daily routines, with significantly lower scores (*p* < 0.05) than Clusters 1, 3, and 4; only Cluster 5 expressed more negative evaluations. Their agreement with sustainability-related statements was also significantly lower than that of Clusters 1 and 3, and comparable to Cluster 4. For perceived contributions to food security, they fell into the lowest-scoring group, along with Cluster 5 (*p* < 0.05). These results reinforce their profile as strongly dairy-oriented consumers with limited recognition of the value of PBDAs.

Cluster 3 (PB-Oriented Dairy Critics), alongside Cluster 1, constitutes the most favourable segment towards PBDAs. They considered PBDAs highly attractive and easy to integrate into daily life, with scores significantly higher (*p* < 0.05) than Clusters 2, 4, and 5. They also perceived PBDAs consumption as unproblematic, with mean scores not significantly different from Cluster 1 and significantly higher than those of the remaining clusters. On all sustainability dimensions—environmental friendliness, climate impact, and food insecurity—they reported the highest levels of endorsement, statistically comparable to Cluster 1 and significantly higher than those of Clusters 2 and 5. 

Cluster 4 (Moderate Dairy Consumers) scores close to the midpoint of the scale in terms of all perceptions of PBDAs. They rated PBDAs as moderately appealing and somewhat compatible with daily eating habits, with scores significantly higher than Clusters 2 and 5 but significantly lower than Clusters 1 and 3 (*p* < 0.05). PBDAs consumption was regarded as relatively unproblematic, and sustainability beliefs were moderate, statistically higher than the most sceptical groups but lower than the most favourable ones. Perceived contributions to food security followed a similar pattern, with scores significantly above those of Clusters 2 and 5 but lower than those of Clusters 1 and 3 (*p* < 0.05).

Cluster 5 (Dairy-Oriented PB-Uninformed) combined pro-dairy attitudes with the lowest awareness of plant-based alternatives. They rated PBDAs as the least appealing and least compatible with their daily eating habits, with mean scores significantly lower (*p* < 0.05) than all other clusters. While they did not perceive PBDAs consumption as especially problematic, their ratings were still significantly lower than those of Clusters 1 and 3. Belief in the environmental, food security, and climate-related benefits of PBDAs was also weakest in this group, with scores significantly lower than those of all other clusters (*p* < 0.05), particularly Clusters 1 and 3, and statistically comparable to Cluster 2. 

### 3.5. Perception of Selected Dairy Alternatives’ Attributes

To characterise how the identified segments differ in their evaluation of plant-based dairy alternatives, respondents rated these products on a series of semantic differential scales. The attributes covered perceived healthiness, environmental favourability, modernity, nutritional value, degree of processing, innovativeness, naturalness, taste, accessibility, and price. All items were assessed on seven-point bipolar scales with higher values indicating a more positive evaluation of the right-hand pole (Table 5).

**Table 5:** Mean values on individual cluster profiles based on semantic differential perceptions.

Items	Total	Cluster 1	Cluster 2	Cluster 3	Cluster 4	Cluster 5
Unhealthy–Healthy	3.99	3.76 a	3.89 a	5.06 b	3.97 a	3.63 a
Unfavourable–Favourable for the environment	4.50	4.88 b	4.39 a	5.28 b	4.28 a	4.14 a
Traditional–Modern	5.23	5.29 b	5.82 c	5.59 bc	4.65 a	5.67 bc
Low–High nutritional value	4.18	4.85 c	3.73 a	4.75 c	4.14 b	3.62 a
Low-processed–Highly processed	4.53	4.52 ab	5.30 c	4.22 a	4.13 a	4.90 b
Non-innovative–Innovative	4.84	5.04 b	5.12 b	5.53 c	4.38 a	4.92 b
Unnatural–Natural	3.88	4.69 d	3.28 b	4.74 d	3.96 c	2.75 a
Unpalatable–Tasty	3.66	3.55 b	3.38 b	4.56 c	3.76 c	3.18 a
Hard to access–Easy to access	5.28	4.57 a	6.20 b	5.81 b	4.78 a	5.84 b
Cheap–Expensive	4.84	4.04 a	5.64 b	5.42 b	4.41 a	5.49 b

Values represent mean scores on a 7-point scale, anchored at “totally disagree” and “totally agree”. Different letters within rows indicate statistically significant differences between groups at *p* < 0.05 based on one-way ANOVA with Scheffé post hoc tests.

Cluster 1 (Defensive but Informed Dairy Enthusiasts) rated PBDAs moderately to positively across most dimensions. Their perceptions of nutritional value, naturalness, and environmental favourability were significantly higher than those of Clusters 2 and 5 (*p* < 0.05). In contrast, ratings for taste, and healthiness did not differ significantly from those of Clusters 2, indicating a more neutral view on sensory attributes. PBDAs were perceived as less accessible and less expensive than in most other clusters, with statistically lower ratings for accessibility compared to Clusters 2, 3, and 5.

Cluster 2 (Unquestioning Dairy Traditionalists) rated PBDAs less favourably regarding taste, nutritional value, and naturalness, with significantly lower scores than Clusters 1 and 3 (*p* < 0.05), and often similar to Cluster 5. Despite this, they rated PBDAs as the most accessible, most processed, most expensive, and most modern, significantly higher than Clusters 1 and 4.

Cluster 3 (PB-Oriented Dairy Critics) presented the most consistently positive perception of PBDAs across all dimensions. Cluster 5 (Dairy-Oriented PB-Uninformed) rated taste and naturalness significantly lower than all other clusters (*p* < 0.05), and their ratings of nutritional value and environmental favourability were significantly lower than those of the more PBDA-oriented segments (particularly Clusters 1 and 3). In terms of perceived healthiness, they did not differ significantly from Clusters 1, 2, and 4. Additionally, they perceived PBDAs as less processed than Clusters 2 and 5, and as highly accessible, while innovativeness ratings were higher than those reported in Clusters 2 and 5. While they acknowledged PBDAs as relatively expensive, this did not diminish their overall positive evaluation.

Cluster 4 (Moderate Dairy Consumers) rated PBDAs as moderately healthy and natural, with healthiness scores similar to those of Clusters 1, 2, and 5 but clearly lower than Cluster 3. Their perceptions of taste, nutritional value, and naturalness were significantly more positive than in Cluster 5, while remaining below those observed in Clusters 1 and 3 for nutritional value and naturalness. For taste, Cluster 4 reported significantly more favourable evaluations than Clusters 1, 2, and 5 and similar ratings to Cluster 3. They perceived PBDAs as least innovative (significantly lower than Clusters 1–3 and 5) and least modern, yet did not see them as highly processed. Ratings for accessibility and price were statistically comparable to Cluster 1.

Cluster 5 (Dairy-Oriented PB-Uninformed) rated taste and naturalness significantly lower than all other clusters (*p* < 0.05), and their ratings of nutritional value and environmental favourability were significantly lower than those of the more PBDA-oriented segments (particularly Clusters 1 and 3). On perceived healthiness, they did not differ significantly from Clusters 1, 2, and 4. PBDAs were simultaneously perceived as modern, innovative, accessible, and highly processed, similar to ratings in Cluster 2.

### 3.6. Consumption of Milk Products and Plant-Based Alternatives per Cluster

Consumption frequency was analysed as an ordinal categorical variable and is presented as category-based distributions (n, %). Accordingly, differences between clusters refer to variations in the proportion of respondents across frequency categories and were assessed using χ^2^ tests based on the full distributions (Table 6).

In the total sample, the majority of respondents reported consuming conventional dairy products, such as cow’s milk, fermented dairy drinks, and curd/cottage cheeses, at least once per week, with substantial shares indicating consumption several times a week or daily. In contrast, plant-based milk, yoghurt/kefir, and cheese alternatives remained niche products, with non-users constituting the largest category in all three cases, and regular use (defined as weekly or more frequent consumption) limited to a minority of respondents.

All associations between cluster membership and the frequency of product consumption were statistically significant for each category (Pearson’s χ^2^ tests, all *p* < 0.001). Overall, the five segments varied more in their use of plant-based dairy alternatives than in their consumption of conventional dairy.

Cluster 1 (Defensive but Informed Dairy Enthusiasts) and Cluster 2 (Unquestioning Dairy Traditionalists) were the most dairy-oriented segments. Both clusters showed higher proportions of respondents reporting weekly or more frequent consumption of milk, fermented dairy drinks, and curd/cottage cheese compared with the remaining clusters. However, Cluster 1 also reported relatively frequent use of plant-based alternatives, particularly plant-based milk and yoghurt, resulting in a dual-consumption profile.

The distribution of PBDAs frequency categories in Cluster 1 was similar to that in Cluster 3 (PB-Oriented Dairy Critics), with both clusters showing higher proportions in the weekly-or-more categories and lower proportions of non-users compared to the other clusters. At the same time, Cluster 3’s intake of conventional dairy was slightly lower than that of Clusters 1 and 2, indicating partial substitution of dairy with plant-based alternatives.

Cluster 4 (Moderate Dairy Consumers) resembled the overall sample in terms of traditional dairy intake and showed limited but noticeable experimentation with PBDAs. Occasional use (1–3 times per month or less) was more prevalent than in the most sceptical clusters (Cluster 2 and Cluster 5), although weekly or more frequent PBDA consumption remained relatively rare. 

Cluster 5 (Dairy-Oriented PB-Uninformed) combined high-frequency consumption of conventional dairy products, particularly milk and curd/cottage cheeses, with very limited engagement with plant-based alternatives. In this cluster, the majority of respondents reported never consuming plant-based milk, yoghurt/kefir, or cheese alternatives, and regular PBDAs use was nearly absent.

### 3.7. Trial Intentions and Information Needs Related to Plant-Based Dairy Alternatives

To complement the attitudinal and behavioural aspects, an additional set of items assessed under what conditions respondents would be willing to try plant-based milk and dairy alternatives. Three statements addressed, respectively, a general intention to taste PBDAs before purchase, interest in trying free samples, and the desire to obtain preparation information prior to buying. All items were rated on seven-point agreement scales, and their cluster-specific means are presented in Table 7.

Cluster 1 (Defensive but Informed Dairy Enthusiasts) showed the strongest trial intentions across all measures. They were significantly more likely than all other clusters to express willingness to try free samples (*p* < 0.05) and obtain preparation information (*p* < 0.05). Their intention to taste PBDAs before purchase was also significantly higher than in Clusters 4 and 5 (*p* < 0.05), and comparable to Clusters 2 and 3. This group demonstrated the most proactive, information-seeking attitude toward trying PBDAs.

Cluster 2 (Unquestioning Dairy Traditionalists) expressed a relatively strong willingness to taste PBDAs, with scores significantly above Cluster 4 (*p* < 0.05), but did not differ significantly from other clusters in their interest in free samples or preparation guidance.

Cluster 3 (PB-Oriented Dairy Critics) reported a similarly high intention to taste PBDAs before purchase, with no significant differences compared to Clusters 1 and 2. However, their interest in free samples and preparation information did not differ significantly from that of most other clusters, indicating a trial-friendly orientation that is not strongly reliant on external support.

Cluster 4 (Moderate Dairy Consumers) exhibited the lowest overall motivation to try PBDAs before purchase. Their scores on this item were significantly lower than those reported by Clusters 1–3 (*p* < 0.05). For free samples and preparation information, however, their responses did not differ significantly from those of Clusters 2, 3, and 5.

Cluster 5 (Dairy-Oriented PB-Uninformed) occupied an intermediate position. Their intention to taste PBDAs before purchase was significantly lower than that of Cluster 1 (*p* < 0.05) but did not differ significantly from Clusters 2 and 3, and was higher than that of Cluster 4. Their responses regarding free samples and preparation information did not differ significantly from those in Clusters 2–4, indicating limited but not absent trial readiness.

## 4. Discussion

The present study identified five distinct consumer segments that systematically differed in their pro-dairy justifications, avoidance of dairy products due to their animal origin, and familiarity with PBDAs. The findings reveal a diverse consumer landscape where plant-based products must compete with established dairy consumption habits influenced by differing beliefs, product perceptions, consumption patterns, and willingness to try PBDAs. These results concur with previous research indicating that attitudes towards cow’s milk and its plant-based alternatives are shaped by a complex interplay of factors—including health concerns, environmental awareness, animal welfare considerations, and cultural traditions—that differ significantly among consumer groups [31,41,44,45,46]. Despite the growing popularity of plant-based alternatives, most consumers still prefer traditional dairy because they perceive a “need” for it, which includes nutritional and habitual reasons [4].

### 4.1. Motivations and Barriers in Adopting Plant-Based Dairy Alternatives

A comparison of cluster profiles highlights key motivators and barriers that influence the adoption of plant-based dairy alternatives. As seen in numerous studies, concerns related to health, the environment, and animal welfare emerge as primary positive drivers, particularly among segments that are PBDA-friendly. Clusters 1 and 3 perceive PBDAs as clearly beneficial for the environment, considering these products healthier and more “natural” than the sceptical groups do. This aligns with findings that animal welfare, sustainability, and personal health are top reasons consumers choose plant-based milks [41,44,47].

Ethical motives are especially strong among younger demographics: studies indicate that young adults view consuming PBDAs as a way to act on their pro-environmental values and concern for animals [10,31,48]. In the sample, younger and more educated respondents were indeed more common in the pro-PBDAs clusters (1 and 3) than in the traditionalist Cluster 2. This supports the idea that increased sustainability awareness and nutrition knowledge predispose consumers to try dairy alternatives.

Another motivation evident in the results is curiosity and openness to innovation. The strong interest in product sampling observed within Cluster 1 indicates that even consumers with a clear preference for traditional dairy might be receptive to appealing alternatives, especially when supported by effective marketing strategies or social influence. This finding aligns with qualitative research conducted in Poland, Germany, and France, which identified curiosity, health awareness, and peer influence as key drivers of initial engagement with dairy substitutes [41].

However, several barriers still hold back wider adoption of PBDAs, as seen in the sceptical Clusters 2 and 5 (and to a lesser degree, Cluster 4). One significant obstacle is sensory appeal—the taste, texture, and overall palatability of plant-based alternatives compared to traditional dairy products. In our study, both Clusters 2 and 5 viewed PBDAs as less appealing in flavour and less “tasty” or satisfying than dairy, particularly Cluster 5, and to a lesser extent Cluster 2. Previous research consistently highlights the taste of PBDAs as a crucial factor in their adoption [28,34,49,50,51]. Even Cluster 1, despite its overall positive evaluation of PBDAs, rated their taste relatively moderate, indicating that further enhancements in flavour and texture remain necessary. In contrast, Cluster 2—the most resistant group—described plant-based alternatives as overly processed and less enjoyable than conventional dairy. This perception aligns with previous research reporting that many consumers associate plant-based cheeses and milks with undesirable textures or “off-flavours” [52]. Overcoming the sensory barrier is crucial, as pleasant taste and mouthfeel are key to repeat purchases.

Habit and convenience constitute another major barrier. Dairy products benefit from an established advantage—they are deeply woven into daily routines and food culture [53]. By contrast, seeking out plant-based substitutes can feel inconvenient or even socially awkward (e.g., asking for non-dairy milk when it is not the norm) [54]. Cluster 2 exemplifies this inertia: dairy is linked to tradition, comfort, and normalcy, yielding a resistance to change [4]. Even Cluster 4, while somewhat receptive to PBDAs, shows only irregular use of these alternatives. The perceived inconvenience of PBDAs also affects cooking—for example, some consumers are unsure how to incorporate plant-based cheese into recipes, or they find that plant milks do not behave like cow’s milk in certain dishes. Indeed, plant-based milks often do not perform like cow’s milk in culinary applications due to differences in protein content, fat distribution, and stability [28,51].

Such practical hurdles can discourage all but the most determined individuals. Over time, as PBDAs become more common in restaurants, cafés, and household routines, this barrier is expected to diminish. The dual-consumption pattern observed in Cluster 1 indicates that even habitual dairy consumers can adopt alternatives into their routines.

Price and affordability also emerged as significant barriers. Many consumers perceive plant-based alternatives as costly luxury items. In the Finnish interviews, even those aware of dairy’s issues admitted that budget constraints and the lower price of cow’s milk often “overruled” their conscience [54]. In Poland, where price sensitivity is high, this is a critical factor—a previous Polish study found widespread dissatisfaction with the higher prices of plant milks compared to cow’s milk [40]. Although price perceptions were not a central to the segmentation, the tendency of Cluster 5 to perceive plant-based dairy alternatives as relatively expensive suggests that cost may contribute to their unfavourable image. Similarly, Cluster 4 viewed plant-based alternatives as “not excessively expensive” yet simultaneously “least innovative”, suggesting scepticism regarding their value for money. These findings emphasise the importance of economic factors in influencing consumer decisions; even environmentally conscious individuals might continue to buy dairy products if plant-based options are seen as less affordable. Targeted policies and market strategies—such as subsidies, price promotions, or local sourcing to lower production costs—may therefore be necessary to boost PBDAs adoption among more price-sensitive consumer groups.

Another obstacle is knowledge and trust—many consumers have doubts about the nutritional value, ingredients, and processing of PBDAs. This was evident in Clusters 2 and 5, which questioned the healthiness and “naturalness” of plant alternatives, often describing them as highly processed or unnatural products. These opinions reflect the findings of Adamczyk et al. [41], who found that focus group participants frequently questioned whether plant-based substitutes were truly healthy or merely “ultra-processed” imitations. Low familiarity with PBDAs, particularly evident in Cluster 5, likely contributes to scepticism regarding their healthfulness and authenticity. The most pro-PBDAs segments—Clusters 1 and 3—also showed higher levels of product familiarity, indicating that greater self-reported familiarity is linked to more favourable assessments of dairy alternatives. Limited self-reported familiarity can undermine the perceived benefits of PBDAs and reinforce the belief that they are “trendy but over-processed, low-value options”, while the image of traditional dairy as natural and wholesome continues to dominate. Addressing such misconceptions through education is therefore important. Clear labelling and marketing that highlight natural ingredients may help rebuild consumer trust. At the same time, reformulation of more highly processed alternatives is necessary to prevent their association with ultra-processed foods.

### 4.2. Psychological Factors Differentiating the Clusters: Moral Dissonance and Avoidance Mechanisms

By integrating justification, avoidance, and perceived familiarity, this study extends prior PBDA research beyond single-factor explanations, providing a more psychologically grounded account of why adoption remains selective and segment-specific. The identified clusters varied not only in attitudes and perceptions but also in how they cope with the moral and cognitive dissonance surrounding animal-based foods. The results confirm the role of psychological defence mechanisms in maintaining or rejecting dairy consumption. “Defensive but Informed Dairy Enthusiasts” (Cluster 1) are committed to dairy, but demonstrate psychological defensiveness—actively avoiding information on animal exploitation and dairy production, despite being relatively familiar with PBDAs. This group appears to embody a tension between familiarity and attachment—they are familiar with the arguments for alternatives (scoring high on PBDAs familiarity) and hold positive perceptions of many PBDAs attributes, yet they remain emotionally attached to dairy. Interestingly, Cluster 1 respondents showed the highest trial intentions for new PBDA products despite their strong psychological defensiveness toward dairy production. This aligns with the idea that making alternatives convenient and trialable could help them overcome their resistance to introducing PBDAs in their diets. This segment reflects a coping strategy known as strategic dissociation, as observed in studies examining cognitive dissonance in food ethics [55,56]. Indeed, research on the “meat paradox” has shown that consumers often cope with guilt by avoiding information and dissociating meat from its animal origins [57]. The findings of this study reveal a similar paradox related to dairy, described as the “dairy paradox,” where people feel conflicted about the ethical and environmental issues associated with dairy but continue to consume it [55]. Cluster 3 (PB-Oriented Dairy Critics) fits that pattern: they have low attachment to dairy, acknowledge animal welfare problems, and show little need for avoidance or self-justification. Unlike Clusters 1, they rarely rely on avoidance strategies, aligning their consumption with moral beliefs. Cluster 3, although the smallest group, may represent a growing number of idealists who could influence dietary changes if their example spreads.

In contrast, Cluster 2 “Unquestioning dairy traditionalists” exhibited almost no signs of internal conflict. Rather than avoiding the issue, they deny that any ethical problem with dairy exists. This reflects a moral disengagement strategy, where the downplaying or denial of animal minds and pain is used to justify continued use. They also emphasise dairy’s necessity and tastiness, classic rationalisations for prolonging the status quo. Prior studies confirm that denial (“animals don’t really suffer”) and justifications of necessity or normalcy are common to neutralise guilt [58]. Cluster 5 “Dairy-attached but PB-uninformed” seems to experience neither active dissonance nor active defence—instead, they exhibit low awareness of plant-based options and issues. They agree with pro-dairy sentiments and lean toward Cluster 2’s sceptical view of PBDAs’ benefits, yet they have the lowest familiarity with alternatives by far. This indicates ignorance (information deficiency) as a factor: many within this group may simply not have access to alternatives or relevant debates. Their resistance is more based on habit and lack of knowledge rather than explicit ideological arguments.

Conversely, the more open segments (Clusters 1, 3, and 4) indicate that exposure and familiarity can lessen the psychological barriers. Cluster 1, despite their defensiveness, is familiar with PBDAs and even uses them on occasion. Cluster 3, with the highest familiarity and least dissonance, shows that a consistent pro-PBDAs attitude is possible when ethical/environmental motives align with behaviour. The “Unquestioning Dairy Traditionalists” (Cluster 2) exhibit the most unambiguous pro-dairy segment. They express high endorsement of dairy’s nutritional and hedonic value, while denying animal suffering. These consumers exhibit low PBDAs familiarity and represent the cultural mainstream in countries with strong dairy traditions [4]. “Moderate Dairy Consumers” (Cluster 4) occupy intermediate positions across all three dimensions. Their behaviour tends to be situationally driven and context-sensitive, reflecting ambivalent or transitional attitudes [59].

### 4.3. Implication for Communication Strategies

The findings emphasise that encouraging dietary shifts towards more plant-based dairy within the Polish dairy-centric context requires segment-specific, psychologically informed communication strategies. For the more PBDAs-oriented and informed segments (Clusters 1 and 3), messaging and product positioning may build on ethical and environmental motives while supporting routine integration through practical, use-case oriented guidance (e.g., how products can be incorporated into everyday meals). For moderate and ambivalent consumers (Cluster 4), strategies that reduce perceived risk and effort—such as low-threshold trial opportunities and reassurance regarding taste, processing, and everyday compatibility—may be particularly important. For dairy-attached and less familiar consumers (especially Cluster 5), interventions should prioritise accessible, non-confrontational education that clarifies what PBDAs are and how they can be used, while avoiding identity-threatening framing and positioning PBDAs as a complementary rather than a substitutive option.

### 4.4. Limitations and Direction for Future Research

This study has several limitations that should be taken into account when interpreting the findings. First, the cross-sectional design does not permit causal inferences regarding the relationships between psychological factors and the adoption of plant-based dairy alternatives. Second, all measures relied on self-reported data, which may be affected by recall bias and social desirability bias.

An additional limitation relates to the CAWI technique used in this study. Although internet access is widespread in Poland, individuals with limited digital access or lower digital literacy—particularly among older age groups—may be underrepresented. Accordingly, the results should be interpreted primarily as representative of the online adult population, and generalisation to fully offline populations should be made with caution.

Importantly, this study was conducted in Poland, a country with a strong dairy tradition and deeply embedded dairy consumption habits. Consequently, the identified segments—and their relative prevalence—may reflect features specific to dairy-focused food cultures and should not be indiscriminately generalised to countries with lower dairy consumption or more mature plant-based markets. Cross-national comparative research is required to determine whether similar psychological segments appear across different cultural and dietary contexts.

It should also be noted that familiarity with plant-based dairy alternatives was measured as self-reported perceived familiarity rather than objective nutritional knowledge, which may not fully capture consumers’ actual understanding of product composition or nutritional adequacy.

A further limitation concerns the operationalisation of consumers’ information needs related to plant-based dairy alternatives. In this study, information needs were measured narrowly as preparation- and usage-oriented guidance intended to facilitate initial product trial. Nutrition-related information needs, such as label comprehension and understanding of nutrient fortification (e.g., calcium, vitamin B12, iodine), were not directly assessed. Given the substantial variability in nutritional composition and fortification practices across PBDA product categories, perceived nutritional adequacy may represent a distinct and influential dimension of consumer decision-making that is not captured by the present measures.

Future research should build on these findings by employing longitudinal or experimental designs to test whether the identified psychological segments are associated with sustained PBDAs adoption over time and to clarify temporal relationships between pro-dairy justifications, avoidance of animal-origin considerations, perceived familiarity, and consumption behaviour. Cross-national comparative studies would further aid in assessing the robustness and cultural specificity of the segment structure and its prevalence beyond dairy-centric contexts such as Poland. Finally, considering the clear differences at the category level observed in this study, future research could benefit from examining PBDA adoption separately for different product categories and from supplementing self-reported measures with behavioural or objective indicators to enhance inference.

## 5. Conclusions

This study identified five psychologically distinct consumer segments differing in pro-dairy justifications, avoidance of animal-origin considerations, and self-reported familiarity with plant-based dairy alternatives. These psychological profiles were systematically associated with differences in PBDAs-related beliefs, product perceptions, consumption patterns, and trial intentions, demonstrating substantial heterogeneity in how PBDAs are perceived and incorporated within a dairy-centric food culture. Taken together, the findings underline that consumer responses to plant-based dairy alternatives are shaped not only by attitudes toward PBDAs themselves, but also by underlying attachment to dairy and the psychological strategies used to maintain or challenge existing dietary practices. This highlights the value of segmentation approaches that integrate defence mechanisms and knowledge-related factors when analysing transitions toward more plant-based diets. From an applied perspective, the results underscore the need for differentiated, segment-sensitive strategies rather than uniform communication or product approaches. Coordinated efforts by industry, communication stakeholders, and policymakers, tailored to distinct consumer profiles, may help reduce psychological and practical barriers and facilitate consumer-relevant shifts towards more plant-based dietary patterns in dairy-centric contexts.

## Figures and Tables

**Table 1:** Factor analysis on segmenting variables.

Item	Factor 1	Factor 2	Factor 3
We need milk and dairy products for healthy nutrition.	0.830		
Dairy products are too tasty to worry about what critics say.	0.822		
I enjoy eating/drinking dairy so much that I could never give it up.	0.794		
Animals don’t really suffer when they are used for milk production.	0.713		
Milk and dairy products are produced in a way that minimises animal pain and discomfort.	0.545		
I know the advantages and disadvantages of plant-based alternatives to animal products.		0.832	
I know what plant-based alternatives to animal products mean.		0.807	
I know why the production of plant-based alternatives to animal products should be developed.		0.777	
When I look at dairy products, I try not to associate them with animals.			0.752
I don’t like to think about where the dairy products I consume come from.			0.680
I try not to think about what happens in the dairy industry.			0.663
I would have a problem visiting a dairy farm.			0.586

**Table 2:** Socio-demographic profile of clusters.

Category	Total 1220	Cluster 1 (212) 17.4%	Cluster 2 (226) 18.5%	Cluster 3 (139) 11.4%	Cluster 4 (470) 38.5%	Cluster 5 (173) 14.2%
Gender						
Female	(617) 50.6%	(114) 53.6%	(97) 42.9%	(92) 65.7%	(251) 53.5%	(64) 37.0%
Male	(603) 49.4%	(98) 46.4%	(129) 57.1%	(48) 34.3%	(219) 46.5%	(109) 63.0%
18–24	(116) 9.5%	(25) 11.8%	(12) 5.3%	(18) 12.8%	(55) 11.7%	(6) 3.5%
25–34	(217) 17.8%	(50) 23.7%	(20) 8.8%	(31) 22.0%	(102) 21.7%	(14) 8.1%
35–44	(287) 23.5%	(51) 24.2%	(32) 14.1%	(38) 27.0%	(124) 26.4%	(42) 24.4%
45–54	(250) 20.5%	(31) 14.7%	(61) 26.9%	(21) 14.9%	(86) 18.3%	(51) 29.7%
55–64	(211) 17.3%	(39) 18.5%	(50) 22.0%	(19) 13.5%	(66) 14.0%	(37) 21.5%
65–70	(140) 11.5%	(15) 7.1%	(52) 22.9%	(14) 9.9%	(37) 7.9%	(22) 12.8%
Education						
Primary	(90) 7.4%	(15) 7.1%	(12) 5.3%	(10) 7.2%	(36) 7.7%	(17) 9.8%
Vocational (basic)	(321) 26.3%	(59) 27.8%	(55) 24.3%	(14) 10.1%	(138) 29.4%	(55) 31.6%
Secondary (general/technical)	(463) 37.9%	(82) 38.7%	(96) 42.5%	(54) 38.8%	(178) 37.9%	(53) 30.5%
Higher (Bachelor/Engineer/Master)	(347) 28.4%	(56) 26.4%	(63) 27.9%	(61) 43.9%	(118) 25.1%	(49) 28.2%
Place of living						
Rural area	(487) 40.0%	(88) 41.7%	(91) 40.1%	(54) 39.1%	(188) 40.1%	(66) 38.2%
Town ≤ 20,000	(159) 13.1%	(28) 13.3%	(32) 14.1%	(10) 7.2%	(61) 13.0%	(28) 16.2%
Town 20,000–50,000	(130) 10.7%	(22) 10.4%	(21) 9.3%	(18) 13.0%	(44) 9.4%	(25) 14.5%
City 50,000–200,000	(204) 16.7%	(32) 15.2%	(41) 18.1%	(18) 13.0%	(87) 18.6%	(26) 15.0%
City > 200,000	(238) 19.5%	(41) 19.4%	(42) 18.5%	(38) 27.5%	(89) 19.0%	(28) 16.2%
Refused to answer	(159) 13.1%	(23) 10.9%	(21) 9.3%	(22) 15.8%	(68) 14.5%	(25) 14.5%
We live very poorly—insufficient even for basic needs	(49) 4.0%	(11) 5.2%	(4) 1.8%	(5) 3.6%	(26) 5.5%	(3) 1.7%
We live modestly—we must economise a lot	(163) 13.3%	(38) 17.9%	(25) 11.0%	(11) 7.9%	(64) 13.6%	(25) 14.5%
We live average—enough for daily needs; must save for bigger purchases	(726) 59.4%	(112) 52.8%	(136) 59.9%	(83) 59.3%	(282) 59.9%	(113) 65.3%
We live well—without significant savings	(228) 18.6%	(44) 20.8%	(54) 23.8%	(31) 22.1%	(71) 15.1%	(28) 16.2%
We live very well—we can afford some luxuries	(17) 1.4%	(2) 0.9%	(4) 1.8%	(3) 2.1%	(8) 1.7%	(0) 0.0%
No opinion	(40) 3.3%	(5) 2.4%	(4) 1.8%	(7) 5.0%	(20) 4.2%	(4) 2.3%

**Table 3:** Cluster means for segmenting variables: pro-dairy justifications, familiarity with plant-based alternatives, and dissociation/avoidance.

Item	Total	Cluster 1	Cluster 2	Cluster 3	Cluster 4	Cluster 5
We need milk and dairy products for a healthy diet.	5.20	5.84 c	6.57 d	3.88 a	4.41 b	5.81 c
Dairy products are too tasty to worry about what critics say.	4.77	5.56 c	6.08 d	2.99 a	4.07 b	5.40 c
I enjoy eating/drinking dairy so much that I could never give it up.	4.78	5.61 d	6.25 e	3.21 a	4.03 b	5.15 c
Animals don’t really suffer when they are used for milk production.	4.75	5.61 cd	5.86 d	3.28 a	4.10 b	5.22 c
Milk and dairy products are produced in a way that minimises animal pain and discomfort.	4.42	5.38 d	4.98 c	3.75 a	3.95 ab	4.31 b
I know the advantages and disadvantages of plant-based alternatives to animal products.	4.23	5.43 d	4.98 c	5.21 cd	3.81 b	2.14 a
I know what plant-based alternatives to animal products mean.	4.62	5.63 c	5.66 c	5.87 c	4.07 b	2.55 a
I know why the production of plant-based alternatives to animal products should be developed.	4.05	5.36 c	3.95 b	5.39 c	3.81 b	2.15 a
When I look at dairy products, I try not to associate them with animals.	3.95	5.71 d	2.52 a	3.16 b	3.96 c	4.33 c
I don’t like to think about where the dairy products I consume come from.	4.20	5.67 d	3.37 a	3.26 a	4.06 b	4.63 c
I try not to think about what happens in the dairy industry.	4.45	5.83 d	3.94 b	3.47 a	4.27 bc	4.70 c
I would have a problem visiting a dairy farm.	3.10	4.69 d	1.36 a	3.11 c	3.56 c	2.19 b

Values represent mean scores on a 7-point scale, anchored at “totally disagree” and “totally agree”. Different letters within rows indicate statistically significant differences between groups at *p* < 0.05 based on one-way ANOVA with Scheffé post hoc tests.

**Table 4:** Consumer beliefs about plant-based milk and dairy alternatives by cluster.

Items	Total	Cluster 1	Cluster 2	Cluster 3	Cluster 4	Cluster 5
Plant-based milk and dairy alternatives are attractive for me daily	3.36	4.68 d	2.69 b	4.46 d	3.57 c	2.01 a
Plant-based milk and dairy alternatives fit well into my daily eating habits	3.25	4.57 d	2.66 b	4.41 d	3.54 c	1.93 a
Consumption of plant-based milk and dairy alternatives is unproblematic	4.31	4.91 c	4.00 b	4.90 c	3.97 b	3.44 a
Plant-based milk and dairy alternatives are environmentally friendly	4.35	5.07 c	3.97 b	5.05 c	4.22 b	3.64 a
Plant-based milk and dairy alternatives help reduce food insecurity	4.15	4.96 c	3.57 a	4.70 c	4.01 b	3.49 a
Plant-based milk and dairy alternatives help combat climate change	3.98	4.84 c	3.38 a	4.90 c	4.01 b	3.12 a

Values represent mean scores on a 7-point scale, anchored at “totally disagree” and “totally agree”. Different letters within rows indicate statistically significant differences between groups at *p* < 0.05 based on one-way ANOVA with Scheffé post hoc tests.

**Table 6:** Declared frequency of dairy and plant-based alternatives consumption by cluster.

Product	Frequency	Total n (%)	Cluster 1 n (%)	Cluster 2 n (%)	Cluster 3 n (%)	Cluster 4 n (%)	Cluster 5 n (%)
Milk	Never	113 (9.3)	14 (6.6)	17 (7.5)	21 (15.1)	45 (9.6)	16 (9.2)
	1–3 times per month or less	215 (17.6)	30 (14.2)	16 (7.0)	26 (18.7)	106 (22.6)	37 (21.4)
	Once a week	199 (16.3)	29 (13.7)	32 (14.1)	22 (15.8)	92 (19.6)	24 (13.9)
	Several times a week	331 (27.1)	66 (31.1)	65 (28.6)	26 (18.7)	121 (25.7)	53 (30.6)
	Once a day or more	363 (29.7)	73 (34.4)	97 (42.7)	44 (31.7)	106 (22.6)	43 (24.9)
Fermented dairy drinks	Never	98 (8.0)	15 (7.1)	6 (2.6)	15 (10.7)	46 (9.8)	16 (9.2)
	1–3 times per month or less	280 (22.9)	36 (17.0)	35 (15.4)	31 (22.1)	128 (27.3)	50 (28.7)
	Once a week	288 (23.6)	46 (21.7)	47 (20.7)	37 (26.4)	117 (24.9)	41 (23.6)
	Several times a week	408 (33.4)	75 (35.4)	101 (44.5)	39 (27.9)	139 (29.6)	54 (31.0)
	Once a day or more	148 (12.1)	40 (18.9)	38 (16.7)	18 (12.9)	39 (8.3)	13 (7.5)
Curd/cottage cheeses and desserts	Never	52 (4.3)	5 (2.4)	5 (2.2)	13 (9.4)	26 (5.5)	3 (1.7)
	1–3 times per month or less	306 (25.1)	35 (16.6)	45 (19.8)	28 (20.3)	144 (30.6)	54 (31.0)
	Once a week	353 (28.9)	47 (22.3)	63 (27.8)	38 (27.5)	146 (31.1)	59 (33.9)
	Several times a week	413 (33.9)	90 (42.7)	92 (40.5)	51 (37.0)	133 (28.3)	47 (27.0)
	Once a day or more	96 (7.9)	34 (16.1)	22 (9.7)	8 (5.8)	21 (4.5)	11 (6.3)
Plant-based milk alternatives	Never	630 (51.6)	79 (37.1)	136 (59.9)	35 (25.0)	245 (52.2)	135 (78.0)
	1–3 times per month or less	286 (23.4)	45 (21.1)	48 (21.1)	50 (35.7)	113 (24.1)	30 (17.3)
	Once a week	105 (8.6)	23 (10.8)	19 (8.4)	9 (6.4)	52 (11.1)	2 (1.2)
	Several times a week	128 (10.5)	40 (18.8)	16 (7.0)	25 (17.9)	43 (9.2)	4 (2.3)
	Once a day or more	73 (6.0)	26 (12.2)	8 (3.5)	21 (15.0)	16 (3.4)	2 (1.2)
Plant-based yoghurt/kefir alternatives	Never	657 (53.8)	71 (33.5)	153 (67.4)	50 (36.0)	251 (53.4)	132 (76.3)
	1–3 times per month or less	250 (20.5)	43 (20.3)	42 (18.5)	50 (36.0)	95 (20.2)	20 (11.6)
	Once a week	136 (11.1)	32 (15.1)	17 (7.5)	15 (10.8)	58 (12.3)	14 (8.1)
	Several times a week	115 (9.4)	35 (16.5)	10 (4.4)	18 (12.9)	48 (10.2)	4 (2.3)
	Once a day or more	63 (5.2)	31 (14.6)	5 (2.2)	6 (4.3)	18 (3.8)	3 (1.7)
Plant-based cheese alternatives	Never	759 (62.3)	85 (40.3)	159 (70.7)	71 (51.1)	295 (62.8)	149 (86.1)
	1–3 times per month or less	210 (17.2)	40 (19.0)	42 (18.7)	42 (30.2)	71 (15.1)	15 (8.7)
	Once a week	106 (8.7)	33 (15.6)	11 (4.9)	9 (6.5)	48 (10.2)	5 (2.9)
	Several times a week	99 (8.1)	33 (15.6)	8 (3.6)	9 (6.5)	48 (10.2)	1 (0.6)
	Once a day or more	44 (3.6)	20 (9.5)	5 (2.2)	8 (5.8)	8 (1.7)	3 (1.7)

**Table 7:** Conditions for trying plant-based milk and dairy alternatives by cluster.

Items	Total	Cluster 1	Cluster 2	Cluster 3	Cluster 4	Cluster 5
Before I decide to buy plant-based milk and dairy alternatives, I would like to try them first.	4.65	5.14 c	4.77 bc	4.87 bc	4.27 a	4.49 ab
Before I decide to buy plant-based milk and dairy alternatives, I want to try free samples	4.61	5.17 b	4.63 a	4.51 a	4.19 a	4.52 a
Before I decide to buy plant-based milk and dairy alternatives, I want preparation information	4.40	4.87 b	4.37 a	4.49 a	4.05 a	4.08 a

Values represent mean scores on a 7-point scale, anchored at “totally disagree” and “totally agree”. Different letters within rows indicate statistically significant differences between groups at *p* < 0.05 based on one-way ANOVA with Scheffé post hoc tests.

## Data Availability

The data presented in this study are available on request from the corresponding author because the data have not yet been made available in publicly available databases.
